# Zoning of air quality index (PM_10_ and PM_2.5_) by Arc-GIS for Khorramabad city, Iran

**DOI:** 10.1016/j.dib.2018.05.063

**Published:** 2018-05-19

**Authors:** Maryam Kianisadr, Mansour Ghaderpoori, Ali Jafari, Bahram kamarehie, Mohammadamin Karami

**Affiliations:** a Department of Environment, College of Basic Sciences, Hamedan Branch, Islamic Azad University, Hamedan, Iran; b Nutritional Health Research Center and Department of environmental health engineering, School of Health and Nutrition, Lorestan University of medical sciences, Khorramabad, Iran; c Department of environmental health engineering, School of Health and Nutrition, Lorestan University of medical sciences, Khorramabad, Iran

**Keywords:** Air quality index, PM_10_, PM_2.5_, Khorramabad, GIS

## Abstract

Nowadays in many countries, air pollution is one of the major issues affecting human health. Among the various air pollutants particulate matters are mainly present in ambient air pollution. The purpose of this study was to measure the concentration of particulate matter (PM) (namely PM_2.5_ and PM_10_) and to conduct zoning via GIS software in Khorramabad city (Summer – 2017). According to the findings, the average concentrations of PM_2.5_ in July, August and September were 100.1, 116.3, and 199.8 μg/m^3^, respectively. Furthermore, the average concentrations of PM_10_ in July, August and September were 199.8, 215.7, and 190.8 μg/m^3^, respectively. The findings of this study also indicated that due to continuous dust storms,particularly in recent years, the air pollution status in Khorramabad was not suitable that can adversely affect public health.

**Specifications Table**

**Table:** 

Subject area	*Chemistry, biology*
More specific subject area	*Air pollution monitoring and quality*
Type of data	*Table, figure*
How data was acquired	*Sampling (by Environmental Dust Monitor, model: Envirocheck 107) and measuring the concentration of PM*_*10*_*and PM*_*2.5*_*in of Khorramabad city. After determining the concentration, AQI were calculated. Finally, the collected and analyzed data entered the GIS software*
Data format	*Raw, analyzed,*
Experimental features	*According to the city map, 45 stations of air pollution were selected as sampling stations. Until concentration measurement, all samples were stored in standard conditions and were analyzed for thePM*_*10*_*and PM*_*2.5*_
Data source location	*Khorramabad city Iran (33° 48׳ N, 48° 35׳ E), Lorestan province, west of Iran*
Data accessibility	*Data are included in this research and supplemented excel file*

**Value of the data**

• In recent years, dust storms, in Iran and especially in west of the country, have increased significantly. As a result, the continuous monitoring and presenting the major pollutants is important.
• According to previous studies, particulates (PM_2.5_ and PM_10_) are the main sources of airborne diseases for public health.
• Particulate mattes can carry toxic pollutants such as heavy metals and organic compounds. Therefore, their continuous monitoring is very necessary.
• AQI shows the impact of air pollution on health. This index is provided by United States Environmental Protection Agency 2003.

## 1 Data

This study measured the concentration of particulate matters (PM_2.5_ and PM_10_) in Khorramabad city and conducted its zoning via GIS software and IDW method.

## 2 Experimental design, materials, and methods

**Fig. 1:**
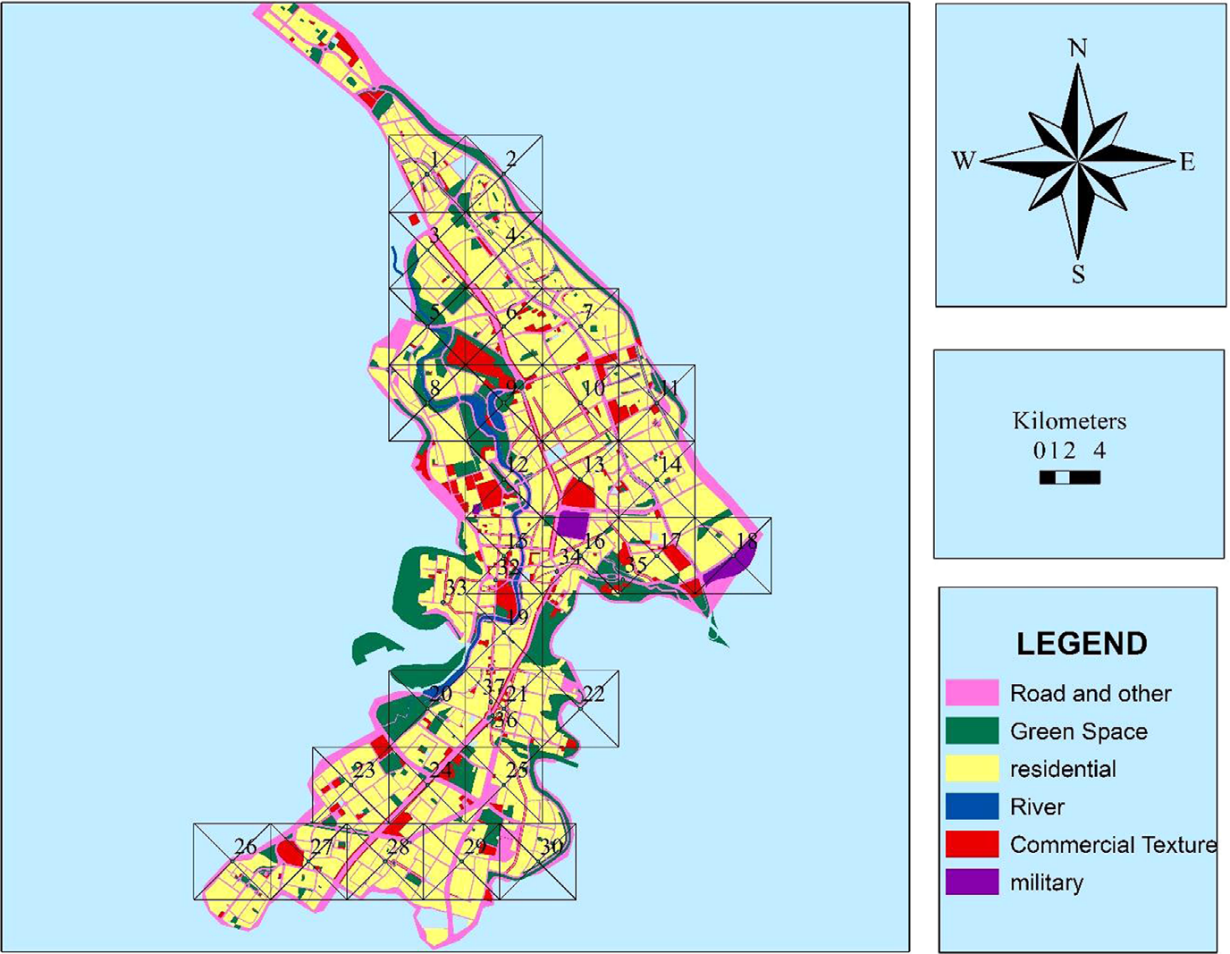
The location of the air pollutant measurement stations in Khorramabad city, Iran.

In order to determine the number of measurement stations in the study area, we used the equation of n = (var^2^ * z^2^)/d^2^. According to this equation, the number of sampling stations was 30. In addition to the 30 stations mentioned above, 8 stations in traffic and crowded areas of the city were also selected for air pollutants measurement. The location of the stations are shown in Fig. 1. Also, due to the fact that IDW method was used to prepare zoning maps of air pollution in GIS, so to increase the accuracy of calculations, 7 stations were added to study stations. As a result, a total of 45 stations were selected. The whole sample was taken in summer season. In this study, PM_10_ and PM_2.5_ were measured by Environmental Dust Monitor. After the measurement, the AQI index was calculated according to Eq. (1):

(1) $$I_{p}=\frac{I_{Hi}-I_{Lo}}{BP_{Hi}-BP_{Lo}}(C_{p}-BP_{Lo})+I_{Lo}$$

**Fig. 2:**
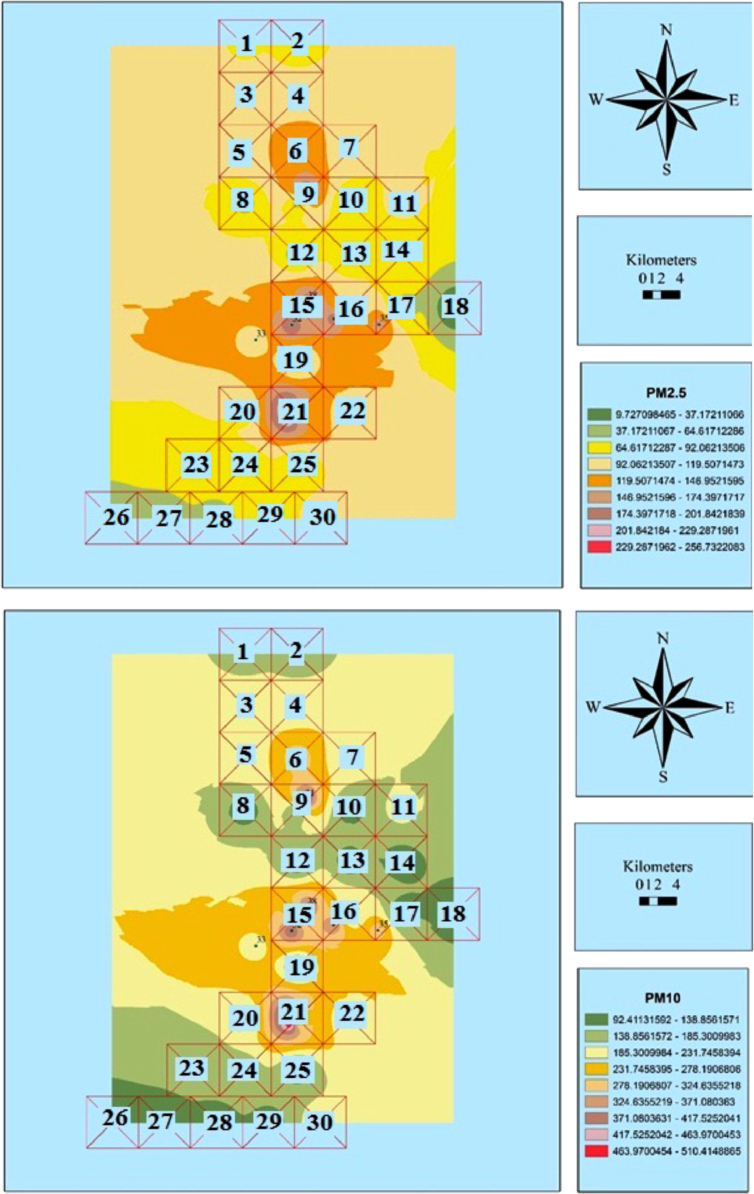
Zoning the distribution of the average concentration of PM_2.5_ and PM_10_ in July using GIS.

**Fig. 3:**
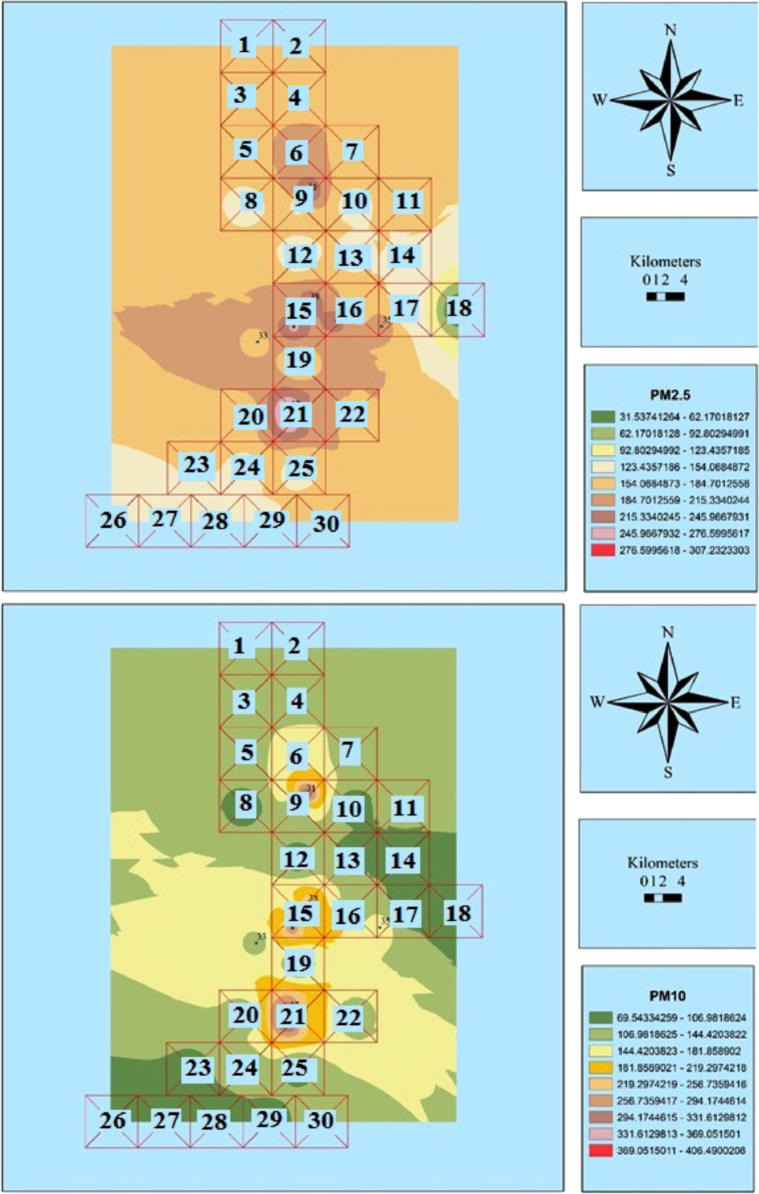
Zoning the AQI distribution for PM_2.5_ and PM_10_ in July using GIS.

**Fig. 4:**
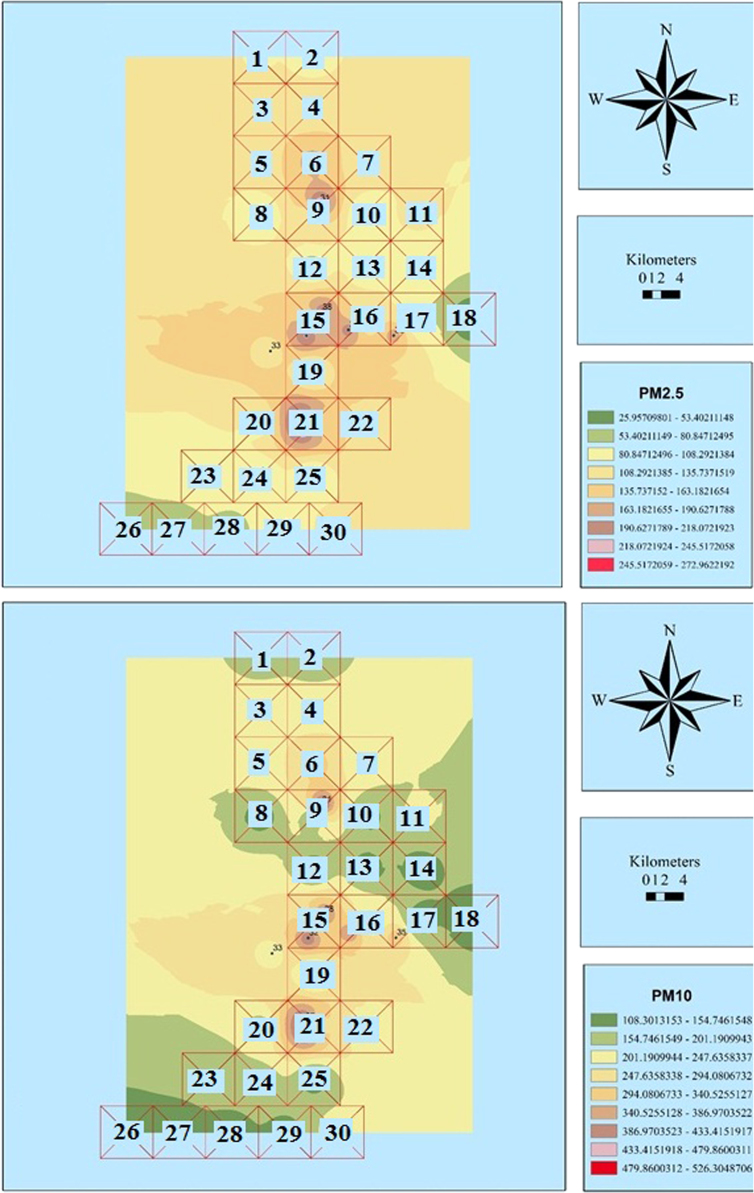
Zoning the distribution of the average concentration of PM_2.5_ and PM_10_ in August using GIS.

**Fig. 5:**
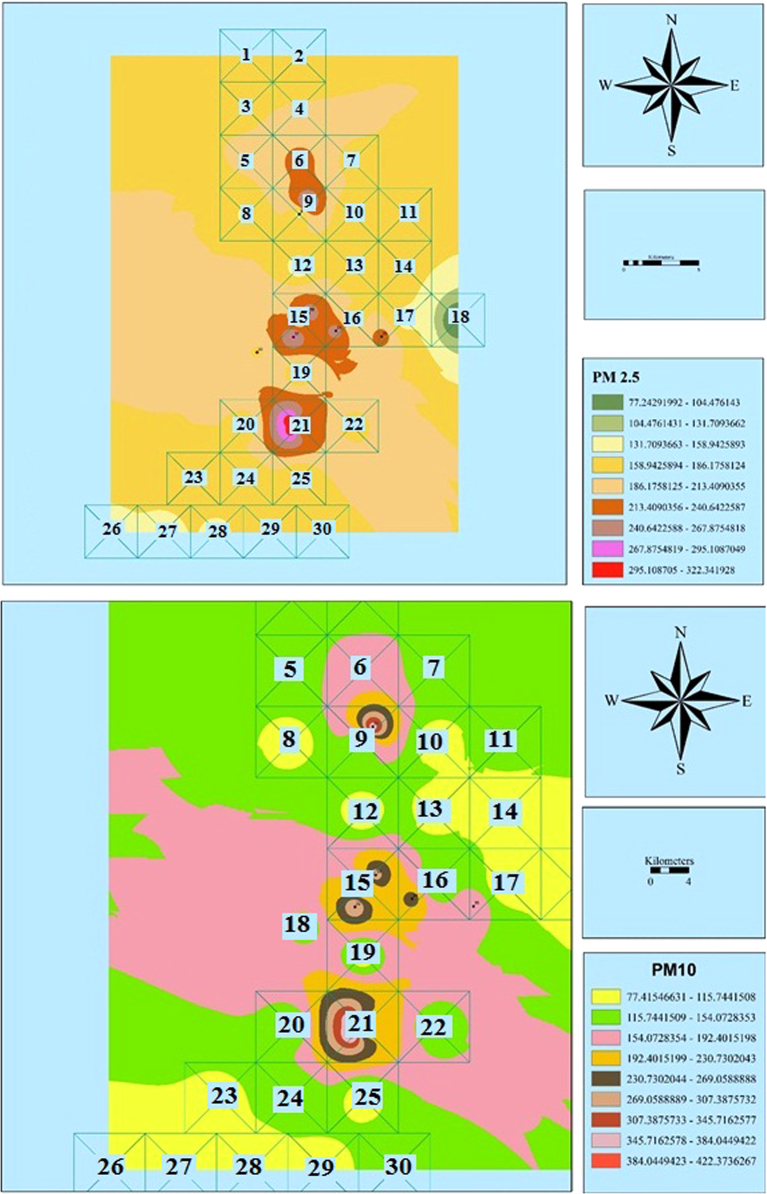
Zoning the AQI distribution for PM_2.5_ and PM_10_ in August using GIS.

**Fig. 6:**
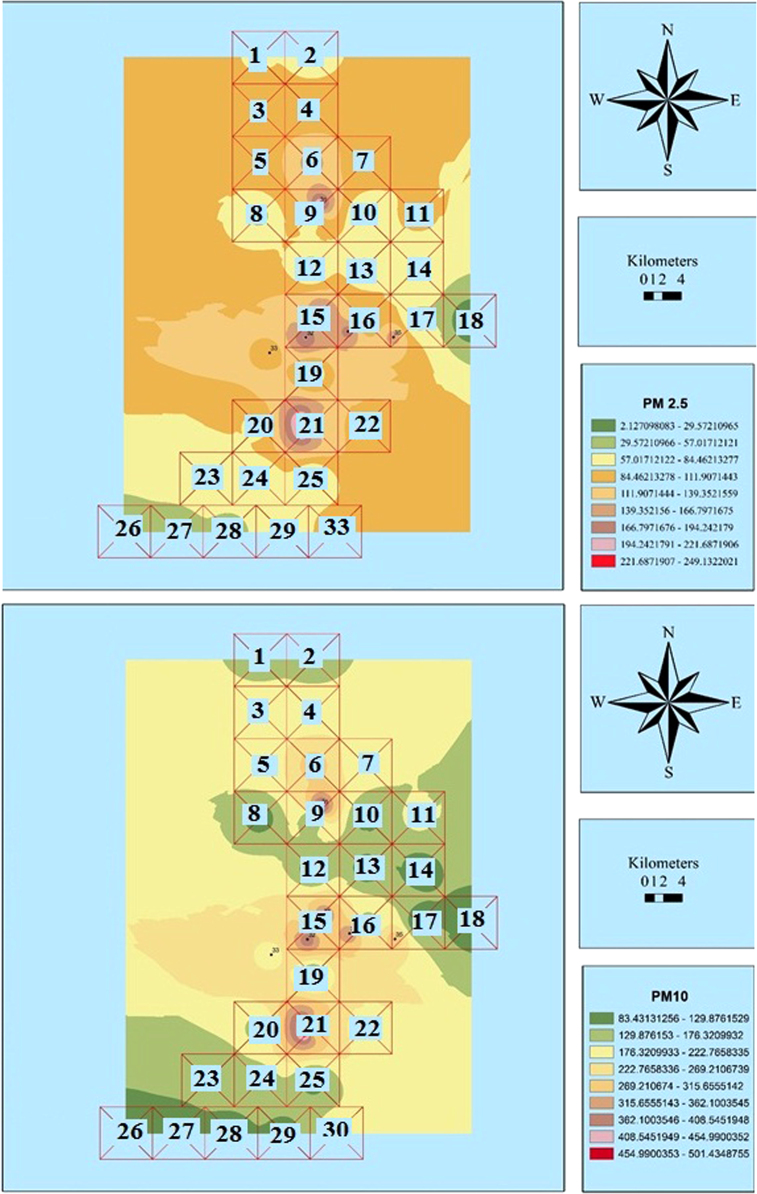
Zoning the distribution of the average concentration of PM_2.5_ and PM_10_ in September using GIS.

**Fig. 7:**
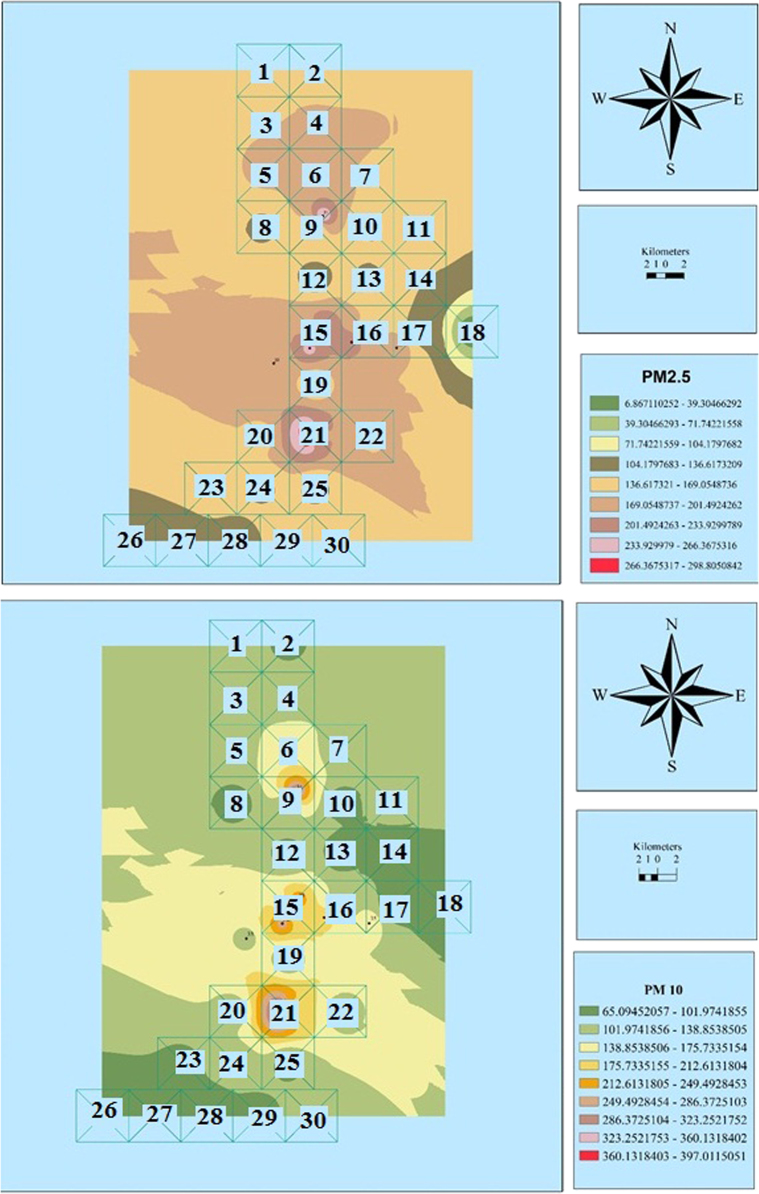
Zoning the AQI distribution for PM_2.5_ and PM_10_ in September using GIS.

The measured concentrations of PM_2.5_ and PM_10_ are shown in Table 1. Also, Fig. 2, Fig. 3, Fig. 4, Fig. 5, Fig. 6, Fig. 7 show the results of the zoning of PMs data using the GIS. The average concentrations of PM_2.5_ in July, August, and September were 100.1, 116.3, and 199.8 μg/m^3^, respectively. The minimum and maximum concentrations of PM_2.5_ in this period were 9.7 and 273.3 μg/m^3^, respectively. The average concentrations of PM_10_ in July, August and September were 199.8, 215.7, and 190.8 μg/m^3^, respectively. The minimum and maximum concentrations of PM_10_ in this period were 83.2 and 526.8 μg/m^3^, respectively. According to the US Environmental Protection Agency, the standard concentrations of PM_2.5_ and PM_10_ are 150 and 65 μg/m^3^, respectively. Unfortunately, the study results showed that the concentration of PM_2.5_ and PM_10_ in the city is worrying [1], [2], [3], [4], [5], [6], [7], [8], [9], [10], [11], [12], [13], [14], [15].

**Table 1:** The measured concentrations of PM_2.5_ and PM_10_ in Khorramabad in summer 2016.

**station**		**1**	**2**	**3**	**4**	**5**	**6**	**7**	**8**	**9**	**10**	**11**	**12**	**13**	**14**	**15**	**16**	**17**	**18**	**19**
	**July**	9.7	45.1	48.8	50.0	51.1	51.7	52.9	53.2	55.1	56.2	56.6	56.8	57.8	64.0	64.8	69.1	72.1	72.9	74.2
**PM**_**2.5**_	**August**	25.9	61.4	65.0	66.3	67.3	67.9	69.1	69.4	71.3	72.4	72.8	73.0	74.1	80.2	81.0	85.3	88.3	89.2	90.4
	**September**	2.1	37.5	41.2	42.4	43.5	44.1	45.3	45.6	47.5	48.6	49.0	49.2	50.2	56.4	57.2	61.5	64.5	65.3	66.6
	**July**	92.2	94.3	98.9	99.2	101.2	107.1	107.4	108.2	109.2	109.8	110.9	111.8	112.1	112.7	141.1	143.2	146.2	148.1	154.2
**PM**_**10**_	**August**	108.1	110.2	114.8	115.1	117.1	123.0	123.3	124.1	125.1	125.7	126.8	127.7	128.0	128.6	157.0	159.1	162.1	164.0	170.1
	**September**	83.2	85.3	89.9	90.3	92.2	98.1	98.5	99.2	100.3	100.8	101.9	102.9	103.1	103.7	132.1	134.2	137.3	139.1	145.3
**Sation**		**20**	**21**	**22**	**23**	**24**	**25**	**26**	**27**	**28**	**29**	**30**	**31**	**32**	**33**	**34**	**35**	**36**	**37**	**38**
	**July**	76.2	88.0	89.2	100.1	103.1	103.9	105.6	107.3	108.2	109.9	111.2	156.9	159.8	187.2	200.1	204.8	223.0	250.1	257.1
**PM**_**2.5**_	**August**	92.5	104.2	105.5	116.4	119.4	120.1	121.8	123.6	124.4	126.1	127.5	173.1	176.0	203.4	216.3	221.0	239.2	266.4	273.3
	**September**	68.6	80.4	81.6	92.5	95.5	96.3	98.0	99.7	100.6	102.3	103.6	149.3	152.2	179.6	192.5	197.2	215.4	242.5	249.5
	**July**	159.1	168.1	178.2	191.2	201.1	201.1	207.2	208.1	209.0	210.2	214.9	301.2	308.2	370.1	400.1	410.0	456.1	480.3	511.0
**PM**_**10**_	**August**	175.0	184.0	194.1	207.1	217.0	217.0	223.1	224.0	224.8	226.1	230.8	317.1	324.1	386.0	416.0	425.9	472.0	496.2	526.9
	**September**	150.1	159.1	169.2	182.3	192.1	192.1	198.2	199.1	200.0	201.3	205.9	292.2	299.2	361.2	391.1	401.0	447.1	471.3	502.0
