# Supplementary material for: Zoning of air quality index (PM10 and PM2.5) by Arc-GIS for Khorramabad city, Iran
Source: Data Brief. 2018 May 19;19:1131–41. doi: 10.1016/j.dib.2018.05.063 (PMC6139470; doi:10.1016/j.dib.2018.05.063)
Supplement: Supplementary file 1 — Supplementary material [file mmc1.docx]

Dear Professor,

Editor of **Data in Brief**

The authors of this article declare that they have no conflict of interests.

**Zoning of Air Quality Index (PM_10_ and PM_2.5_) by Arc-GIS for Khorramabad city, Iran**

Maryam Kianisadr, Mansour Ghaderpoori, Ali Jafari, Bahram kamarehie, Mohammadamin Karami
